# How Placebo Needles Differ From Placebo Pills?

**DOI:** 10.3389/fpsyt.2018.00243

**Published:** 2018-06-05

**Authors:** Younbyoung Chae, Ye-Seul Lee, Paul Enck

**Affiliations:** ^1^Acupuncture and Meridian Science Research Center, College of Korean Medicine, Kyung Hee University, Seoul, South Korea; ^2^Department of Anatomy and Meridians, College of Korean Medicine, Gachon University, Seongnam, South Korea; ^3^Department of Internal Medicine, Psychosomatic Medicine and Psychotherapy, University of Tübingen, Tübingen, Germany

**Keywords:** acupuncture, blinding, control, placebo, physiology

## Abstract

Because acupuncture treatment is defined by the process of needles penetrating the body, placebo needles were originally developed with non-penetrating mechanisms. However, whether placebo needles are valid controls in acupuncture research is subject of an ongoing debate. The present review provides an overview of the characteristics of placebo needles and how they differ from placebo pills in two aspects: (1) physiological response and (2) blinding efficacy. We argue that placebo needles elicit physiological responses similar to real acupuncture and therefore provide similar clinical efficacy. We also demonstrate that this efficacy is further supported by ineffective blinding (even in acupuncture-naïve patients) which may lead to opposite guesses that will further enhances efficacy, as compared to no-treatment, e.g., with waiting list controls. Additionally, the manner in which placebo needles can exhibit therapeutic effects relative to placebo pills include enhanced touch sensations, direct stimulation of the somatosensory system and activation of multiple brain systems. We finally discuss alternative control strategies for the placebo effects in acupuncture therapy.

## Introduction

Acupuncture is a therapeutic intervention performed by “inserting one or more needles into specific sites on the body surface for therapeutic purposes” (1). Placebo needles were developed and validated to evaluate the efficacy of acupuncture treatment in randomized controlled clinical trials (RCTs) (2, 3). Due to the indistinguishably inert nature of placebo controls compared with active treatments, placebo-controlled studies enable determination of the therapeutic effects of target treatments from unspecific treatment effects, such as medical context and consequent expectation. Similarly, placebo needles must be indistinguishable from real acupuncture needles and not produce any physiological therapeutic effects. To achieve this, non-penetrating needles with a similar appearance to real acupuncture needles, which retract telescopically into the needle handle when pressed on the skin, were developed because they provide patients with the visual illusion that their skin is being penetrated, much like a stage dagger in theater performances.

Non-penetrating needles have been commonly used as placebo controls for acupuncture research over several decades (4), and are often seen as standard when investigating the mechanisms underlying the acupuncture effects (5). Interestingly, several studies have shown that the effectiveness of placebo acupuncture needles is similar to that of real acupuncture needles. A systematic review of clinical trials revealed only a small difference between real and placebo needles in terms of pain relief, whereas a moderate difference was found between placebo treatment and no treatment at all, e.g., during a waiting period (6). RCTs have shown that real and placebo acupuncture treatments are equally effective and that both are superior to “treatments as usual” (TAU) for chronic pain (7, 8). Taken together, these findings imply that acupuncture treatment is equally effective as placebo acupuncture and therefore, that acupuncture treatment effects are placebo effects (9). However, the adequacy of the controls being used in these studies remains to be determined (10). Many discussions of whether placebo needles are appropriate controls for acupuncture research have followed the development of these needles (11), and there has been some criticism from a physiological perspective that placebo needles may not be proper controls for acupuncture studies (12). In fact, placebo needles are neither fully indistinguishable from regular needles nor physiologically inert (13, 14). Similarly, a recent meta-analysis suggested that neither the Streitberger device nor the Park Sham device is adequate inert controls for clinical studies (15).

This issue pertains not only to acupuncture needles, but also to other treatment devices that involve physical contact with the patient, such as injections, transcutaneous electrical nerve stimulation, manual therapy, and surgical interventions. Placebo devices, including placebo injections and placebo acupuncture needles, exhibit stronger effects than do oral placebo pills (16). Similarly, a meta-analysis showed that subcutaneous placebo administrations produce greater effects than do oral placebos for the acute treatment of migraine (17). A more recent meta-analysis of the effects of placebo interventions across all clinical conditions showed that physical placebo interventions, including acupuncture, have greater effects than do pill controls (18); sham acupuncture has been shown to have even greater effects than other physical placebos (19). A clinical trial revealed that placebo needles have greater effects than placebo pills on self-reported pain and severity of symptoms in patients with persistent arm pain (20). Expectations on the potential benefit induced in the recipient, influenced by the magnitude of the invasiveness of the intervention, leads to therapeutic effects following a placebo treatment (21). The greater effect of placebo devices compared with placebo pills may be due to the additional physical contact or the tactile component of the intervention, which is minimally present with the use of pharmaceutical pills. Therefore, the contextual effects associated with the preparation of acupuncture treatment devices are multisensory and have a broader impact on the patient. The tactile context of treatment devices such as during acupuncture is essential for the establishment of therapeutic effects (22). In contrast to the use of oral placebo pills, this context has two components: physiological action and ineffective blinding, which initially takes effect once the treatment is applied, and which, therefore, is different from the gradual unblinding due to experiences of adverse events during the drug applications.

Thus, the purpose of the present article was to review the two components of placebo devices, physiological action and effective blinding, and to discuss how these features result in stronger placebo effects relative to oral pills.

## Physiological actions of placebo needles

### The “specific” effect of placebo needles due to tactile stimulation

Pharmaceutical research involving a placebo requires a verum preparation with a specific drug and a placebo preparation without that drug, with the difference in the effects of these two preparations indicating the effectiveness of the target drug. The aims of this type of study design are to exclude any other possible factor that might influence the general effects of medical treatment, such as natural history, regression to the mean, and/or methodological biases, and to test the “true” therapeutic effects of the novel compound (23). Additionally, the non-specific effects of the treatment can be observed by comparing the response with placebo to a no-treatment control condition, e.g., a waiting list; these effects are caused by the treatment preparation itself within a medical context, i.e., the attention the patient receives. The context provided by the medical setting may be referred to as the “specific” effect of the placebo (24). In fact, placebo effects are regarded as brain–body responses to contextual information that promote health and well-being (24).

In the case of placebo needles, tactile stimulation is an additional component that is associated with the treatment context of acupuncture, which is absent in a pharmaceutical context. Due to this component, the expected difference in effect between placebo needle treatment and waiting list groups includes a tactile context that has been overlooked in previous studies. The tactile context provided by the placebo needles, much like the medical context under which a pill is given, cannot be physiologically inert, and this stimulation can even exert similar therapeutic actions by enhancing touch sensations in the body (25). Furthermore, the touch of the placebo needles experienced by the patient initiates a multisensory process and thereby activates bodily self-awareness. Overall, tactile stimulation provides a broader range of contexts that contribute to the effect and improve the healing process relative to other placebo interventions (26). The effect of the tactile component on the patient can be categorized accordingly into sensory-discriminative and affective-social aspects. These aspects of the tactile component play important roles in the therapeutic effect of acupuncture treatment in clinical practice (22), which is examined in the context of placebo needles in the following sections.

### The sensory-discriminative aspect of the touch component of placebo needles

**Figure 1:**
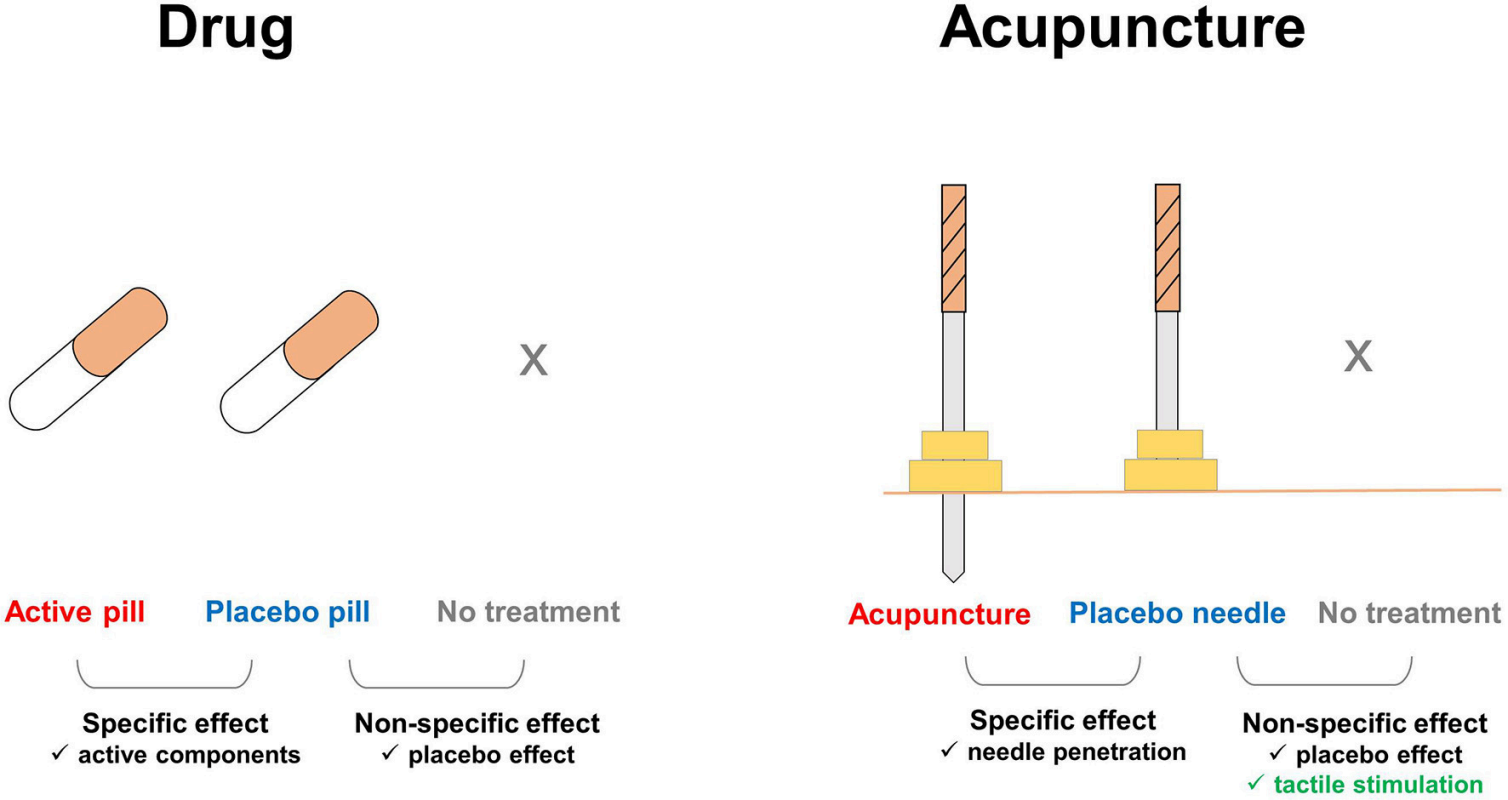
Additional components involved in the effects of placebo needles. In pharmaceutical trials, the nonspecific effects of treatments can be ruled out by comparing the placebo pill group with an untreated group, e.g., on a waiting list. In acupuncture trials, tactile stimulation is an additional factor that affects the placebo needle and untreated groups. Enhanced touch sensations, which are distinct during acupuncture treatment, but absent with placebo pills, remain substantial during placebo needle administration. Thus, placebo needles not only play a role as a cue for treatment expectations, but also evoke the somatosensory system and directly activate multiple brain systems.

Several studies have examined in depth the sensory-discriminative aspect of acupuncture needles. The process of needle insertion and the types of needle manipulation (27) activate diverse touch perception processes and stimulate mechanically sensitive pain fibers (28). This tactile stimulation process produces what is known as the *de qi* sensation (a combination of various sensations that include heaviness, numbness, soreness, and distention), which is fundamental for the therapeutic outcome of acupuncture treatment (29, 30). Placebo needles were first validated as a sufficient control in acupuncture studies under the assumption that a lesser degree of *de qi* sensation would be evoked, thereby leading to less effective clinical outcomes (2, 3). In the initial validation studies of placebo needles, participants were not able to distinguish the placebo needles from real needles, but they experienced a greater degree of *de qi* sensation with real needles than with placebo needles (2, 3, 31) (Figure 1).

On the other hand, a recent validation study of the Streitberger needle conducted with a large population showed no significant difference in *de qi* sensation between patients treated with real and placebo needles, even though the placebo needle does not penetrate the skin (32). Additionally, a study investigating Park Sham devices revealed that the *de qi* sensation induced by real and placebo needles is not distinguishable (33). *De qi* sensation, a composite of unique sensations produced during acupuncture, has been considered to be one of the essential components for clinical efficacy (22). Considering the lack of a significant difference between treatments administered with real and placebo needles, we can assume that the placebo needle exerts an action that is similar to those exerted during real acupuncture.

The somatosensory system is activated directly by placebo needles, which exert various physiological actions in the body that are similar to those exerted by real acupuncture needles. Real and placebo needles produce enhanced skin conductance responses and decrease the heart rate, suggesting that placebo needles are not physiologically inert in terms of autonomic response patterns (14). Furthermore, these autonomic responses to placebo needles might be derived from the patient's orienting responses, or bodily self-awareness (34). A functional magnetic resonance imaging study demonstrated that tactile stimulation, which mimics acupuncture stimulation, not only induces activation in sensorimotor processing regions and deactivation in default-mode network regions, but also modulates higher cognitive areas in the brain (35). Additionally, a meta-analysis of brain imaging studies showed that placebo needles produce weaker, but similar, patterns of brain activation compared with real acupuncture (36). When the placebo needle touches the skin and evokes activity in cutaneous afferent nerves, it seems to act in the brain and result in a limbic touch response (37).

In the pharmaceutical trials, active pills have “true” therapeutic effects of the novel compound in the capsules while placebo pills use the same types of capsules without active components. Placebo pills, of course, can induce tactile sensation on the tongue, but it is not likely that such tactile sensation can be related with the therapeutic effects in the trials. On the other hand, placebo needles can induce tactile sensations around the acupoints that is similar to real acupuncture needles; these tactile sensations themselves could produce physiological actions through the body in the acupuncture trials.

### The affective-social aspect of the touch component of placebo needles

The process of treatment with placebo needles involves a component of touch between the patient and the practitioner. This affective-social aspect, involving slow gentle touch stimulation, activates unmyelinated C tactile fibers (CT afferents) and induces feelings of calm and well-being (38, 39). Prior to inserting and stimulating the needle, the practitioner touches the patient to assess the skin tissue and identify the region to which the needle will be applied. This process of gently touching the patient's skin activates CT afferents and alleviates unpleasantness. Furthermore, this type of pleasant touch reestablishes the patient's sense of self-esteem and well-being by inducing a limbic touch response (39). A clinical study (40) supports the role of affective-social touch in treatments with acupuncture and placebo needles because the enhanced patient–doctor relationship produced greater improvements in patients with irritable bowel syndrome. Additionally, the entirety of the procedure, including warmth, empathy, and the communication of positive expectations, might influence clinical outcomes (40).

Gentle touch, which is always a component of acupuncture treatment, plays a crucial role in the overall outcome of the medical treatment. Gentle touch by a nurse before a surgical operation decreases subjective and objective levels of stress in the patient (41). Furthermore, gentle touch plays a direct moderating role in the physiological responses of the patient such that it lowers blood pressure, enhances transient sympathetic reflexes, and increases pain thresholds (42). The affective-social components of gentle touch also enhance the patient–doctor relationship, even when patients are treated with placebo needles (40). Although the gentle touch component prior to the application of real or placebo needles is not considered to be part of the active component of placebo treatment, it is nevertheless part of the placebo preparation in a clinical acupuncture trial. Thus, compared with the effects observed in a waiting list group or a group receiving another placebo intervention, this component generates a stronger doctor–patient relationship and enhances the placebo effect.

Although the placebo needle acts as a control due to its non-penetrating qualities, the tactile component is not completely removed; thus, its application in acupuncture trials may additionally produce crucial effects such as directly evoking the somatosensory system, strengthening the doctor–patient relationship, and enhancing the patient's general condition. The biophysical effects of placebo needles influence the patient's expectations and contextualization, which likely also play roles in his or her cognitive perception during the treatment process regarding the alleviation of symptoms.

## Blinding of placebo needle applications

### The blinding components of placebo needles

Placebo needles were developed based on a visual illusion that induces the belief that one's skin has been penetrated (2, 3). The tip of the placebo needle is blunt and retracts into the needle's handle; thus, a placebo needle has a shape similar to that of a real needle, but is dissimilar in that it does not penetrate the skin. Because the placebo needle induces the sensation of pricking and appears to penetrate the skin, the patient is more likely to classify placebo needle treatment as active relative to placebo pills. Placebo pills are indistinguishable in appearance from the active drug, but the patient must be convinced that they are receiving real treatment. The chance of determining whether a pill is a placebo or an active treatment is theoretically equal in pharmaceutical trials due to the indistinguishable appearance, smell, and taste of placebo pill compared to active drugs; in contrast, the chance of determining whether a needle is placebo or real is not completely equal, since the patient receiving the treatment while looking at and feeling the needle would be inclined to believe that the placebo treatment is active. Consequently, the probability of a patient determining placebo and real needle would be even more biased, if they have prior experience of acupuncture needling and have felt its therapeutic effects.

Blinding is another important issue that can minimize bias or the potential effect of context on the outcomes of RCTs (43). The blinding index (BI) was developed to assess the success of blinding in clinical trials (44) and is interpreted as a “correct guess beyond chance.” For example, a BI of 1 indicates that all guesses are correct, a BI of −1 indicates that all guesses are incorrect, and a BI of 0 indicates that the probabilities of correct and incorrect guesses are equal (45). When classifying the blinding results of trials, BI values > 0.2 are considered to indicate failed blinding because more participants guessed correctly, BI values < 0.2 and > −0.2 are considered to be random guesses, and BI values < −0.2 are also considered to indicate failed blinding because more participants guessed incorrectly (45). An assessment of blinding in trials involving pharmacological interventions for psychiatric disorders yielded average BI values of 0.18 and 0 in the active treatment and placebo control groups, respectively (46). This finding implies that blinding was established successfully, which is an ideal result from a scientific perspective.

In contrast, people more often respond to placebo needles because they are more likely to believe that they are receiving active treatment, which is also known as an opposite guess (15, 46). Although a recent systematic review of the use of placebo needles for acupuncture in clinical trials with limited reporting of the credibility of blinding showed that participant blinding was successful in most cases (15), participants were less likely than chance levels to believe that the needles were real, rather than placebos. When a BI calculation was applied to this review, the average BI values were 0.55 and −0.33 for the real and placebo needle groups, respectively (15), indicating unsuccessful blinding. Additionally, based on the classification rules for blinding scenarios, 86% of studies have involved unblinded participants in the real acupuncture group (BI > 0.2) and participants making opposite guesses in the placebo group (BI < −0.2) (15).

**Figure 2:**
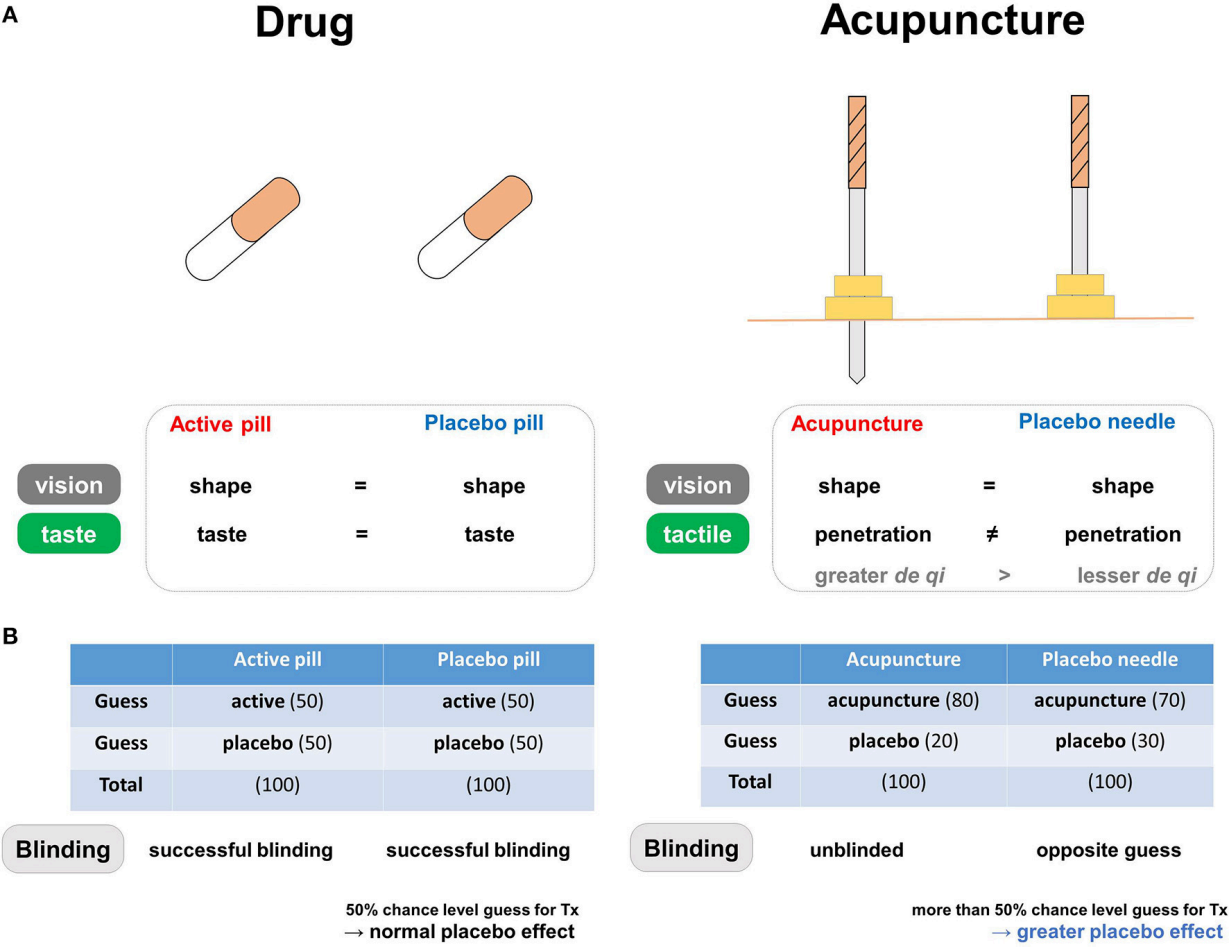
The blinding components of placebo needles**. (A)** Differences in blinding characteristics between placebo needles and placebo pills. In pharmaceutical trials, the similar shapes and tastes of the active and placebo pills prevent patients from correctly guessing whether they are in the treatment group. In acupuncture trials, placebo needles are similar to real acupuncture devices in terms of shape, but not in terms of penetration when applied to the skin. **(B)** Both active and placebo pills have a 50% chance level of being perceived as active in the pharmaceutical trials, whereas both real and placebo acupuncture causes a tendency to believe that they are receiving active treatment in the acupuncture trials. Differences in blinding scenarios for placebo needles and placebo pills. In pharmaceutical trials, successful blinding in the treatment and placebo groups results in patients making random guesses about whether they are receiving active or placebo pills. Acupuncture trials involve different blinding scenarios: “unblinded participants” in the real acupuncture group and participants making “opposite guesses” in the placebo needle group. Due to this unique pattern of blinding, individuals more often respond to placebo needles because they are more likely to believe they are receiving active treatment (i.e., opposite guess).

A recent acupuncture study showed that 61 and 68% of patients administered real and placebo treatments, respectively, perceived treatment type correctly, which implies that blinding was unsuccessful (47). One possible reason for this unsuccessful blinding is the experience of the *de qi* sensation, which could contribute to the correct identification of the treatment (47), even though placebo needling sessions produce substantial levels of this sensation. Another possible explanation is that smaller insertion and pullout forces are used during placebo needling (13). Differences in biomedical forces may be a crucial reason for the association of different somatosensory processes with the use of real and placebo needles (7) (Figure 2A).

### Greater expectations during placebo needling produced greater placebo effects

According to systematic reviews of the BI in clinical trials, pharmacological placebo pills have an approximately 50% chance of being perceived as active, whereas this assumption is not necessarily true for placebo needles (15, 46). While in the aforementioned studies the adverse events of drug trials indicate the risk of unblinding, the BI index seem to have been uncompromised, possibly due to the occurrence time and the frequency of such events.

The discussed BI patterns are often thought to indicate adequate blinding, but a greater probability of believing that a placebo is real might be due to wishful thinking rather the well-known psychological preference toward real or better treatment (48). The greater probability of opposite guesses in placebo needle groups may be related to greater expectations regarding symptom alleviation. Placebo effects, or any improvement in the symptoms or physiological condition of an individual receiving a placebo treatment (23), are based largely on the expectation of receiving actual treatment, cued and contextual conditioning, and/or observational and social learning (49). Thus, patients may have higher levels of expectation during placebo needling than when receiving placebo pills, which could contribute to treatment efficacy (50). In this manner, placebo responses may be more frequent in placebo needles than in placebo pills because patients are more likely to perceive the use of placebo needles as active treatment (Figure 2B).

## Alternative control strategies

When blinding becomes difficult (as with sham acupuncture needles) or even impossible (such as with psychotherapy), alternative control strategies are required to separate specific therapy effects from unspecific (e.g., contextual) effects as well as from spontaneous remission and response biases (23). Ineffective or impossible blinding also precludes conventional cross-over designs where each patient serves as his/her own control, thereby reducing the data variance and allowing trials with far less patients than with a parallel-group design. However, cross-over designs carry another risk: that of carry-over effects from one phase to the next. If the carry-over effect is based on Pavlovian conditioning of responses (51), even longer wash-out phases cannot prevent it to occur.

A number of design alternatives have been discussed which all exhibit both specific advantages and pitfalls.

### No treatment controls (NTC)

To separate “spontaneous variation” from “placebo responses”, a “no-treatment” control group appears necessary that determines how much of the unspecific effects can be attributed to spontaneous variation and recovery. Since this is rarely done, the exact size of the contribution of spontaneous variation to the placebo response is known only for minor and benign clinical conditions and may account here for approximately 50% of the placebo effect (52). In experimental settings, “no treatment controls” may also serve to control for habituation and sensitization effects that may occur with repetitive stimulation, e.g. in pain and placebo analgesia experiments.

NTC are limited by ethical rules when patients with a severe clinical condition require treatment and cannot be offered trial participation that would assign them to a NTC group, as set by the Declaration of Helsinki of the World Medical Association (53).

### Waiting list control (WLC), treatment as usual (TAU)

Assigning patients to a “no treatment” group may be ethically problematic, e.g., in case of severe diseases, or when for other reasons the patients require treatment; in such cases WLC and TAU are control strategies for non-drug testing when an inert “placebo” is not available, e.g., in psychotherapy, physical/manual therapy, surgery, and “instrumental” therapies (TENS, transcranial magnetic or direct current stimulation, laser or light therapy), including acupuncture (see above). While some of these therapies have “sham therapy techniques” that can serve as placebo controls, e.g., in acupuncture, others must rely on WLC and TAU as their only control condition.

However, WLC and TAU face significant limitations: while patients expect to receive effective therapy, they are randomized to routine treatment most of them have had in the past (TAU), or (in case of WLC) have to wait for the treatment they were recruited for, resulting in disappointment and potentially nocebo effects (21). This affects only recruitment and compliance, and biases patient populations in such studies.

To avoid WLC and TAU and the associated disadvantages, studies in acute and chronic pain are often conducted comparing a novel drug with another drug already available rather than with placebos (54, 55).

### Comparative effectiveness research (CER)

One approach to circumvent the placebo dilemma in RCT (for ethical as well as for methodological reasons) has recently been favored by drug approval authorities, by boards of medical societies, and by ethics committees, namely to avoid utilization of placebos in clinical trials. Comparative effectiveness research (CER) compares novel treatments to already approved therapies: to the best of our knowledge, this has never been done for acupuncture therapy, e.g., in chronic pain conditions.

However, as has been shown in a number of meta-analyses in depression, schizophrenia. and other diseases, comparing a new therapy to a comparator increases the response solely driven by the higher likelihood of patients to receive active treatments (100%) as compared to placebo-controlled trials (56). In such trials therefore, the placebo response is high but cannot be controlled anymore. Of specific interest is the fact that CER studies need to test for “non-inferiority” of the novel drug, resulting in higher patient numbers (57).

### Cohort multiple randomized controlled trial (CMRCT) design

The “cohort multiple randomized controlled trial” (CMRCT) (58)—formerly also known as the Zelen design (59)—splits the “no treatment” control arm of a drug trial (done for the purpose of mere observation of the natural course of the disease) from the drug trial itself, by recruiting a large cohort of patients for an “observational study” in which patients are followed under their TAU condition.

The observational cohort then serves as the basis for the recruitment of a subsample for the treatment study, either placebo-controlled or CER: patients are randomly approached, but can be selected based on a number of factors accounting for statistical representativeness.

A number of limitations apply, however: “the observational cohort needs to be monitored over time (a cross-sectional sample analysis would not be sufficient to account for changes occurring over time), and it needs to be representative for complete patient cohort affected by the diseases, both in terms of disease features (e.g., symptom severity) as well as disease management (diagnosis, TAU). Once such a cohort it established it may be used for more than one RCT” (21).

## Discussion and conclusion

Similar to other placebo types, placebo needles play an important contextual role in treatment expectations; however, they also directly evoke the somatosensory system and activate multiple brain systems. Placebo preparations are applied in studies to blind participants, and they enable the calculation of chance levels for patients' guesses about whether interventions are therapeutic or inert. However, the probability of making an opposite guess is greater for placebo needles than for placebo pills, which is often explained by patients' greater expectations. Because patients are more likely to perceive placebos as active treatment in placebo needle trials, placebo responses may be observed more frequently to placebo needles than to placebo pills.

The tactile components of acupuncture needle use are crucial factors during treatment preparation and could not be fully controlled for as placebo needles were being developed. The distinctive touch sensations experienced during acupuncture treatment are substantial, even during the administration of placebo needles. Due to the physical contact necessary when applying placebo needles, the validity of these needles as controls has been in question from the perspectives of physiological inertness and blinding. These factors may result in placebo needles exerting stronger placebo effects than do other types of placebo preparation that do not include tactile components. Thus, the development of a technique to control for the tactile components of acupuncture interventions while participants are consciously receiving treatment is an important consideration. The studies reviewed here demonstrated that the *de qi* sensation cannot be completely accounted for when using placebo needles without controlling for the tactile components, which suggests some level of clinical efficacy. Placebo needle administrations may inadvertently, albeit less robustly, activate the somatosensory system and induce regulatory mechanisms that are also triggered by acupuncture needling. Furthermore, placebo needles, or what we have considered to be control needles for experimental studies, may be a form of acupuncture treatment that is low dose or that provides weak stimulation.

In clinical trials, the placebo control should be indistinguishable from the active treatment (i.e., blinding success) and yet physiologically inert (less *deqi* sensation in this case). In the case of acupuncture, however, it is difficult to meet these two criteria simultaneously (60). Most importantly, our argument on the inadequacy of placebo needles as controls in acupuncture trials should not inhibit further acupuncture trials with randomized, controlled designs. Placebo needles indeed are more likely to induce placebo responses than placebo pills, which is largely due to the tactile component that cannot be separated from the components of the real acupuncture needles. In other words, conversely, our arguments imply that acupuncture needles contain a substantial level of placebo effect, which was not completely ruled out by controlling the penetration. It is also important to note that waiting lists do produce unspecific effects on their own (61). Furthermore, recent studies in acupuncture have employed study designs such as pragmatic trials, which compare acupuncture treatment with waiting lists and usual care (62–64), while other innovative control strategies still await validation with acupuncture. In the meantime, the discussion on the effect of the tactile components of placebo needles in its effectiveness as placebos, as well as effective blinding, needs to be continued.

Taken together, the placebo needles do have different characteristics from placebo pills in clinical trials. Our exploration does not imply that acupuncture may be more effective than placebo, but suggests that we have to consider these unique characteristics of placebo needles before we draw premature conclusions that acupuncture itself is just a placebo.

## Author contributions

Conceived and designed the paper: YC and PE. Wrote the first draft of the paper: YC Y-SL, and PE. Revised the paper and approved the final version: YC, Y-SL, and PE.

### Conflict of interest statement

The authors declare that the research was conducted in the absence of any commercial or financial relationships that could be construed as a potential conflict of interest.
